# 4-[1-(4-Hy­droxy-3-meth­oxy­benz­yl)-1*H*-benzimidazol-2-yl]-2-meth­oxy­phenol

**DOI:** 10.1107/S1600536811043935

**Published:** 2011-10-29

**Authors:** Zuo-an Xiao, Tao Gao, Fa-jun Huang, Ting-ting Jiang

**Affiliations:** a School of Chemical Engineering and Food Science, Xiangfan University, Xiangfan 441053, People’s Republic of China

## Abstract

In the title mol­ecule, C_22_H_20_N_2_O_4_, the dihedral angles between the benzimidazole ring system and the benzene rings are 44.26 (2) and 82.91 (2)°. Intra­molecular O—H⋯O hydrogen bonds occur. In the crystal, O—H⋯N and O—H⋯O hydrogen bonds connect the mol­ecules into a two-dimension network parallel to (10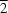) and weak inter­molecular C—H⋯O hydrogen bonds complete the formation of a three-dimensional network.

## Related literature

For the biological appications of benzimidazole compounds, see: Santoro *et al.* (2000 ▶); Sundberg *et al.* (1977 ▶). For related structures, see: Li *et al.* (2005 ▶); Liu *et al.* (2003 ▶); Xi *et al.* (2006 ▶).
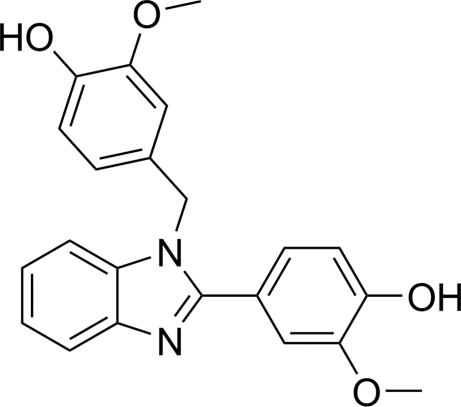


## Experimental

### 

#### Crystal data

C_22_H_20_N_2_O_4_
*M* *_r_* = 376.40
Monoclinic, 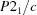

*a* = 7.9717 (9) Å
*b* = 16.4327 (19) Å
*c* = 14.3560 (16) Å
β = 95.133 (2)°
*V* = 1873.0 (4) Å^3^
*Z* = 4
Mo *K*α radiation
μ = 0.09 mm^−1^
*T* = 298 K
0.20 × 0.20 × 0.20 mm

#### Data collection

Bruker SMART CCD diffractometer
Absorption correction: multi-scan (*SADABS*; Sheldrick, 1996 ▶) *T*_min_ = 0.966, *T*_max_ = 0.983
14067 measured reflections
4625 independent reflections
3718 reflections with *I* > 2σ(*I*)
*R*_int_ = 0.064

#### Refinement

*R*[*F* ^2^ > 2σ(*F* ^2^)] = 0.058
*wR*(*F* ^2^) = 0.154
*S* = 1.06
4625 reflections
261 parameters
H atoms treated by a mixture of independent and constrained refinement
Δρ_max_ = 0.30 e Å^−3^
Δρ_min_ = −0.23 e Å^−3^

### 

Data collection: *SMART* (Bruker, 2001 ▶); cell refinement: *SAINT* (Bruker, 2001 ▶); data reduction: *SAINT*; program(s) used to solve structure: *SHELXS97* (Sheldrick, 2008 ▶); program(s) used to refine structure: *SHELXL97* (Sheldrick, 2008 ▶); molecular graphics: *PLATON* (Spek, 2009 ▶); software used to prepare material for publication: *SHELXTL* (Sheldrick, 2008 ▶).

## Supplementary Material

Crystal structure: contains datablock(s) global, I. DOI: 10.1107/S1600536811043935/lh5356sup1.cif

Structure factors: contains datablock(s) I. DOI: 10.1107/S1600536811043935/lh5356Isup2.hkl

Supplementary material file. DOI: 10.1107/S1600536811043935/lh5356Isup3.cml

Additional supplementary materials:  crystallographic information; 3D view; checkCIF report

## Figures and Tables

**Table 1:** Hydrogen-bond geometry (Å, °)

*D*—H⋯*A*	*D*—H	H⋯*A*	*D*⋯*A*	*D*—H⋯*A*
O4—H4*A*⋯O3	0.89 (2)	2.25 (2)	2.6598 (18)	108.0 (18)
O1—H1*A*⋯O2	0.85 (3)	2.22 (3)	2.6631 (18)	113 (2)
O4—H4*A*⋯N2^i^	0.89 (2)	1.90 (2)	2.7671 (18)	165 (2)
O1—H1*A*⋯O4^ii^	0.85 (3)	2.07 (3)	2.7934 (17)	143 (2)
C15—H15*B*⋯O4^iii^	0.97	2.59	3.402 (2)	141

Symmetry codes: (i) 
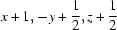
; (ii) 
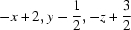
; (iii) 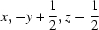.

**Fig. 1.**
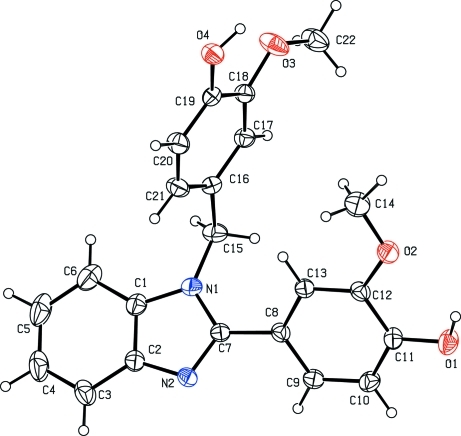
The molecular structure with displacement ellipsoids drawn at the 50% probability level.

**Fig. 2.**
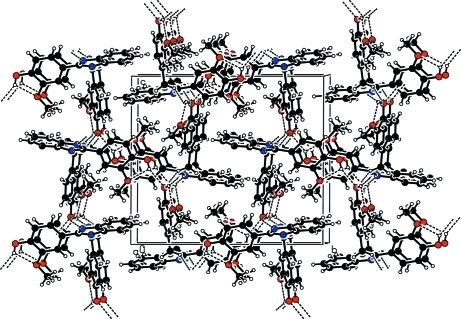
Part of the crystal structure with hydrogen bonds shown as dashed lines.

## supplementary crystallographic information

### Comment

Benzimidazole is a common species in biological and biochemical structures (Sundberg *et al.*, 1977; Santoro *et al.*, 2000). Many benzimidazole derivatives have already been reported (e.g. Liu *et al.*, 2003; Li *et al.*, 2005; Xi *et al.*, 2006) and the preparation of the title compound, (I), is part of our effort to contribute to this research. Herein we report the crystal structure of (I).

In the molecule (Fig. 1) the dihedral angles between the benzimidazole ring system and the benzene rings are [C8-C13] 44.26 (2)° and [C16-C21] 82.91 (2)°. All bond lengths and bond angles are as expected. In the crystal, (Fig.2) molecules are linked by O—H···N and O—H···O and weak C—H···O hydrogen bonds to form a three-dimensional network.

### Experimental

3-Methoxy-4-hydroxyphenyl formaldehyde (10 mmol) and 1,2-diaminobenzene(5 mmol) were mixed in hot water (333 K), the resulting mixture was stirred and refluxed for 3 h at 333 K. The solution was filtered, and the resulting yellow precipitate was recystallized from methanol to obtain pure product. Yellow crystals suitable for an X-ray diffraction study were obtained by slow evaporation of methanol and dimethyl sulfoxide (1:1 *v*/*v*) for two months.

### Refinement

All H atoms were placed in idealized positions [*C*—*H*(methylene)= 0.97 Å, *C*—*H*(methyl)= 0.96 Å and C—H(aromatic)= 0.93 Å] and included in the refinement in a riding-motion approximation, with *U*_iso_(H)=1.5U_eq_ (methyl C) and *U*_iso_(H)=1.2U_eq_ (methylene and aromatic C). Hydrogen atoms bonded to oxygen atoms were located in a difference map and refined freely with U_iso_(H)= 1.5U_eq_(O).

### Crystal data

**Table:**

C_22_H_20_N_2_O_4_	*F*(000) = 792
*M**_r_* = 376.40	*D*_x_ = 1.335 Mg m^−^^3^
Monoclinic, *P*2_1_/*c*	Mo *K*α radiation, λ = 0.71073 Å
Hall symbol: -P 2ybc	Cell parameters from 5118 reflections
*a* = 7.9717 (9) Å	θ = 2.5–27.7°
*b* = 16.4327 (19) Å	µ = 0.09 mm^−^^1^
*c* = 14.3560 (16) Å	*T* = 298 K
β = 95.133 (2)°	Block, yellow
*V* = 1873.0 (4) Å^3^	0.20 × 0.20 × 0.20 mm
*Z* = 4	

### Data collection

**Table:**

Bruker SMART CCD diffractometer	4625 independent reflections
Radiation source: fine-focus sealed tube	3718 reflections with *I* > 2σ(*I*)
graphite	*R*_int_ = 0.064
φ and ω scans	θ_max_ = 28.3°, θ_min_ = 1.9°
Absorption correction: multi-scan (*SADABS*; Sheldrick, 1996)	*h* = −10→9
*T*_min_ = 0.966, *T*_max_ = 0.983	*k* = −21→21
14067 measured reflections	*l* = −17→19

### Refinement

**Table:**

Refinement on *F*^2^	Primary atom site location: structure-invariant direct methods
Least-squares matrix: full	Secondary atom site location: difference Fourier map
*R*[*F*^2^ > 2σ(*F*^2^)] = 0.058	Hydrogen site location: inferred from neighbouring sites
*wR*(*F*^2^) = 0.154	H atoms treated by a mixture of independent and constrained refinement
*S* = 1.06	*w* = 1/[σ^2^(*F*_o_^2^) + (0.0713*P*)^2^ + 0.3997*P*] where *P* = (*F*_o_^2^ + 2*F*_c_^2^)/3
4625 reflections	(Δ/σ)_max_ = 0.001
261 parameters	Δρ_max_ = 0.30 e Å^−^^3^
0 restraints	Δρ_min_ = −0.23 e Å^−^^3^

### Special details

**Table:**

Geometry. All e.s.d.'s (except the e.s.d. in the dihedral angle between two l.s. planes) are estimated using the full covariance matrix. The cell e.s.d.'s are taken into account individually in the estimation of e.s.d.'s in distances, angles and torsion angles; correlations between e.s.d.'s in cell parameters are only used when they are defined by crystal symmetry. An approximate (isotropic) treatment of cell e.s.d.'s is used for estimating e.s.d.'s involving l.s. planes.
Refinement. Refinement of *F*^2^ against ALL reflections. The weighted *R*-factor *wR* and goodness of fit *S* are based on *F*^2^, conventional *R*-factors *R* are based on *F*, with *F* set to zero for negative *F*^2^. The threshold expression of *F*^2^ > σ(*F*^2^) is used only for calculating *R*-factors(gt) *etc*. and is not relevant to the choice of reflections for refinement. *R*-factors based on *F*^2^ are statistically about twice as large as those based on *F*, and *R*- factors based on ALL data will be even larger.

### Fractional atomic coordinates and isotropic or equivalent isotropic displacement parameters (Å^2^)

**Table:**

	*x*	*y*	*z*	*U*_iso_*/*U*_eq_	
C1	0.6594 (2)	0.37261 (10)	0.41671 (11)	0.0434 (4)	
C2	0.4947 (2)	0.34793 (10)	0.39288 (11)	0.0402 (4)	
C3	0.3707 (3)	0.40430 (12)	0.36173 (13)	0.0558 (5)	
H3	0.2600	0.3884	0.3457	0.067*	
C4	0.4192 (4)	0.48403 (13)	0.35583 (16)	0.0709 (7)	
H4	0.3397	0.5228	0.3348	0.085*	
C5	0.5838 (4)	0.50832 (13)	0.38041 (17)	0.0763 (7)	
H5	0.6112	0.5631	0.3757	0.092*	
C6	0.7085 (3)	0.45372 (12)	0.41172 (15)	0.0635 (6)	
H6	0.8186	0.4702	0.4285	0.076*	
C7	0.63537 (19)	0.23907 (9)	0.43023 (10)	0.0346 (3)	
C8	0.67801 (19)	0.15292 (9)	0.44647 (10)	0.0358 (3)	
C9	0.6102 (2)	0.09509 (10)	0.38356 (11)	0.0414 (4)	
H9	0.5444	0.1114	0.3301	0.050*	
C10	0.6401 (2)	0.01332 (10)	0.40007 (12)	0.0448 (4)	
H10	0.5953	−0.0251	0.3572	0.054*	
C11	0.7358 (2)	−0.01188 (10)	0.47960 (11)	0.0398 (4)	
C12	0.8042 (2)	0.04607 (10)	0.54392 (11)	0.0378 (4)	
C13	0.7768 (2)	0.12775 (10)	0.52693 (11)	0.0380 (4)	
H13	0.8239	0.1662	0.5690	0.046*	
C14	0.9622 (3)	0.06781 (14)	0.69039 (15)	0.0690 (6)	
H14A	1.0387	0.1045	0.6640	0.104*	
H14B	1.0213	0.0374	0.7402	0.104*	
H14C	0.8729	0.0983	0.7145	0.104*	
C15	0.9316 (2)	0.29712 (12)	0.45926 (12)	0.0464 (4)	
H15A	0.9819	0.3390	0.4232	0.056*	
H15B	0.9686	0.2449	0.4371	0.056*	
C16	0.9974 (2)	0.30661 (9)	0.56100 (11)	0.0378 (4)	
C17	1.1623 (2)	0.28316 (10)	0.58622 (11)	0.0388 (4)	
H17	1.2285	0.2639	0.5409	0.047*	
C18	1.2289 (2)	0.28829 (10)	0.67832 (11)	0.0378 (3)	
C19	1.1305 (2)	0.31602 (9)	0.74762 (11)	0.0366 (3)	
C20	0.9677 (2)	0.34072 (12)	0.72206 (12)	0.0474 (4)	
H20	0.9015	0.3604	0.7672	0.057*	
C21	0.9017 (2)	0.33641 (12)	0.62925 (13)	0.0480 (4)	
H21	0.7920	0.3538	0.6128	0.058*	
C22	1.4913 (2)	0.22847 (16)	0.65140 (15)	0.0648 (6)	
H22A	1.4335	0.1811	0.6261	0.097*	
H22B	1.5945	0.2124	0.6861	0.097*	
H22C	1.5159	0.2640	0.6013	0.097*	
N1	0.74880 (17)	0.30206 (8)	0.44092 (10)	0.0397 (3)	
N2	0.48213 (17)	0.26436 (8)	0.40272 (9)	0.0383 (3)	
O1	0.76267 (19)	−0.09254 (7)	0.49438 (9)	0.0544 (4)	
H1A	0.804 (3)	−0.1004 (16)	0.550 (2)	0.082*	
O2	0.89395 (17)	0.01375 (7)	0.62064 (9)	0.0531 (3)	
O3	1.38974 (16)	0.26905 (11)	0.71051 (9)	0.0636 (4)	
O4	1.19250 (16)	0.31888 (8)	0.83918 (8)	0.0454 (3)	
H4A	1.291 (3)	0.2940 (14)	0.8496 (17)	0.068*	

### Atomic displacement parameters (Å^2^)

**Table:**

	*U*^11^	*U*^22^	*U*^33^	*U*^12^	*U*^13^	*U*^23^
C1	0.0577 (10)	0.0391 (9)	0.0322 (8)	0.0003 (7)	−0.0019 (7)	−0.0008 (6)
C2	0.0504 (9)	0.0387 (8)	0.0303 (7)	0.0052 (7)	−0.0021 (6)	−0.0002 (6)
C3	0.0647 (12)	0.0525 (11)	0.0487 (10)	0.0180 (9)	−0.0039 (9)	0.0068 (8)
C4	0.1036 (19)	0.0459 (11)	0.0609 (13)	0.0260 (12)	−0.0050 (12)	0.0066 (9)
C5	0.128 (2)	0.0345 (10)	0.0656 (14)	0.0008 (12)	0.0037 (14)	0.0063 (9)
C6	0.0835 (15)	0.0465 (11)	0.0597 (12)	−0.0137 (10)	0.0017 (11)	−0.0027 (9)
C7	0.0386 (8)	0.0384 (8)	0.0260 (7)	0.0014 (6)	−0.0018 (6)	−0.0012 (6)
C8	0.0375 (8)	0.0373 (8)	0.0319 (7)	0.0048 (6)	−0.0003 (6)	0.0000 (6)
C9	0.0462 (9)	0.0437 (9)	0.0324 (8)	0.0032 (7)	−0.0064 (6)	0.0004 (6)
C10	0.0551 (10)	0.0415 (9)	0.0362 (8)	−0.0009 (7)	−0.0046 (7)	−0.0055 (7)
C11	0.0469 (9)	0.0351 (8)	0.0375 (8)	0.0044 (7)	0.0043 (7)	0.0005 (6)
C12	0.0388 (8)	0.0420 (8)	0.0317 (8)	0.0055 (6)	−0.0017 (6)	0.0027 (6)
C13	0.0409 (9)	0.0385 (8)	0.0337 (8)	0.0038 (6)	−0.0030 (6)	−0.0031 (6)
C14	0.0909 (16)	0.0644 (13)	0.0461 (11)	0.0108 (11)	−0.0258 (11)	0.0005 (9)
C15	0.0391 (9)	0.0624 (11)	0.0371 (9)	−0.0039 (7)	0.0004 (7)	−0.0058 (8)
C16	0.0365 (8)	0.0377 (8)	0.0382 (8)	−0.0044 (6)	−0.0021 (6)	−0.0050 (6)
C17	0.0375 (8)	0.0442 (9)	0.0349 (8)	−0.0002 (7)	0.0043 (6)	−0.0060 (6)
C18	0.0344 (8)	0.0421 (8)	0.0365 (8)	0.0009 (6)	0.0006 (6)	−0.0033 (6)
C19	0.0401 (8)	0.0342 (8)	0.0353 (8)	−0.0015 (6)	0.0018 (6)	−0.0065 (6)
C20	0.0415 (9)	0.0594 (11)	0.0414 (9)	0.0091 (8)	0.0049 (7)	−0.0131 (8)
C21	0.0344 (8)	0.0615 (11)	0.0469 (10)	0.0080 (8)	−0.0029 (7)	−0.0096 (8)
C22	0.0434 (11)	0.0933 (16)	0.0573 (12)	0.0176 (10)	0.0016 (9)	−0.0170 (11)
N1	0.0397 (7)	0.0421 (7)	0.0359 (7)	−0.0014 (5)	−0.0052 (5)	−0.0024 (5)
N2	0.0401 (7)	0.0391 (7)	0.0341 (7)	0.0040 (5)	−0.0048 (5)	0.0033 (5)
O1	0.0800 (10)	0.0352 (6)	0.0460 (7)	0.0036 (6)	−0.0051 (7)	0.0024 (5)
O2	0.0673 (8)	0.0450 (7)	0.0432 (7)	0.0066 (6)	−0.0160 (6)	0.0057 (5)
O3	0.0439 (7)	0.1088 (12)	0.0369 (7)	0.0244 (7)	−0.0025 (5)	−0.0126 (7)
O4	0.0447 (7)	0.0573 (8)	0.0335 (6)	0.0082 (5)	0.0000 (5)	−0.0089 (5)

### Geometric parameters (Å, °)

**Table:**

C1—C2	1.388 (2)	C14—O2	1.411 (2)
C1—N1	1.389 (2)	C14—H14A	0.9600
C1—C6	1.393 (3)	C14—H14B	0.9600
C2—N2	1.385 (2)	C14—H14C	0.9600
C2—C3	1.398 (2)	C15—N1	1.460 (2)
C3—C4	1.371 (3)	C15—C16	1.515 (2)
C3—H3	0.9300	C15—H15A	0.9700
C4—C5	1.386 (4)	C15—H15B	0.9700
C4—H4	0.9300	C16—C21	1.384 (2)
C5—C6	1.384 (3)	C16—C17	1.386 (2)
C5—H5	0.9300	C17—C18	1.383 (2)
C6—H6	0.9300	C17—H17	0.9300
C7—N2	1.3171 (19)	C18—O3	1.361 (2)
C7—N1	1.374 (2)	C18—C19	1.397 (2)
C7—C8	1.470 (2)	C19—O4	1.3630 (19)
C8—C9	1.387 (2)	C19—C20	1.378 (2)
C8—C13	1.401 (2)	C20—C21	1.390 (2)
C9—C10	1.381 (2)	C20—H20	0.9300
C9—H9	0.9300	C21—H21	0.9300
C10—C11	1.379 (2)	C22—O3	1.394 (2)
C10—H10	0.9300	C22—H22A	0.9600
C11—O1	1.3564 (19)	C22—H22B	0.9600
C11—C12	1.402 (2)	C22—H22C	0.9600
C12—O2	1.3659 (18)	O1—H1A	0.85 (3)
C12—C13	1.378 (2)	O4—H4A	0.89 (2)
C13—H13	0.9300		
			
C2—C1—N1	105.72 (14)	O2—C14—H14C	109.5
C2—C1—C6	122.08 (17)	H14A—C14—H14C	109.5
N1—C1—C6	132.19 (18)	H14B—C14—H14C	109.5
N2—C2—C1	109.87 (14)	N1—C15—C16	114.96 (14)
N2—C2—C3	129.36 (17)	N1—C15—H15A	108.5
C1—C2—C3	120.74 (17)	C16—C15—H15A	108.5
C4—C3—C2	117.3 (2)	N1—C15—H15B	108.5
C4—C3—H3	121.4	C16—C15—H15B	108.5
C2—C3—H3	121.4	H15A—C15—H15B	107.5
C3—C4—C5	121.7 (2)	C21—C16—C17	118.88 (15)
C3—C4—H4	119.2	C21—C16—C15	123.63 (15)
C5—C4—H4	119.2	C17—C16—C15	117.49 (15)
C6—C5—C4	122.1 (2)	C18—C17—C16	120.46 (15)
C6—C5—H5	118.9	C18—C17—H17	119.8
C4—C5—H5	118.9	C16—C17—H17	119.8
C5—C6—C1	116.1 (2)	O3—C18—C17	125.46 (15)
C5—C6—H6	121.9	O3—C18—C19	113.94 (14)
C1—C6—H6	121.9	C17—C18—C19	120.59 (14)
N2—C7—N1	112.29 (14)	O4—C19—C20	119.87 (15)
N2—C7—C8	123.14 (14)	O4—C19—C18	121.33 (14)
N1—C7—C8	124.57 (14)	C20—C19—C18	118.80 (15)
C9—C8—C13	119.48 (15)	C19—C20—C21	120.45 (16)
C9—C8—C7	119.06 (13)	C19—C20—H20	119.8
C13—C8—C7	121.35 (14)	C21—C20—H20	119.8
C10—C9—C8	120.24 (14)	C16—C21—C20	120.77 (15)
C10—C9—H9	119.9	C16—C21—H21	119.6
C8—C9—H9	119.9	C20—C21—H21	119.6
C11—C10—C9	120.55 (15)	O3—C22—H22A	109.5
C11—C10—H10	119.7	O3—C22—H22B	109.5
C9—C10—H10	119.7	H22A—C22—H22B	109.5
O1—C11—C10	119.40 (15)	O3—C22—H22C	109.5
O1—C11—C12	120.92 (14)	H22A—C22—H22C	109.5
C10—C11—C12	119.67 (15)	H22B—C22—H22C	109.5
O2—C12—C13	125.79 (14)	C7—N1—C1	106.44 (13)
O2—C12—C11	114.31 (14)	C7—N1—C15	127.93 (14)
C13—C12—C11	119.90 (14)	C1—N1—C15	124.98 (14)
C12—C13—C8	120.15 (14)	C7—N2—C2	105.67 (13)
C12—C13—H13	119.9	C11—O1—H1A	109.8 (18)
C8—C13—H13	119.9	C12—O2—C14	117.93 (14)
O2—C14—H14A	109.5	C18—O3—C22	119.05 (14)
O2—C14—H14B	109.5	C19—O4—H4A	112.7 (16)
H14A—C14—H14B	109.5		

### Hydrogen-bond geometry (Å, °)

**Table:**

*D*—H···*A*	*D*—H	H···*A*	*D*···*A*	*D*—H···*A*
O4—H4A···O3	0.89 (2)	2.25 (2)	2.6598 (18)	108.0 (18)
O1—H1A···O2	0.85 (3)	2.22 (3)	2.6631 (18)	113 (2)
O4—H4A···N2^i^	0.89 (2)	1.90 (2)	2.7671 (18)	165 (2)
O1—H1A···O4^ii^	0.85 (3)	2.07 (3)	2.7934 (17)	143 (2)
C15—H15B···O4^iii^	0.97	2.59	3.402 (2)	141.

Symmetry codes: (i) *x*+1, −*y*+1/2, *z*+1/2; (ii) −*x*+2, *y*−1/2, −*z*+3/2; (iii) *x*, −*y*+1/2, *z*−1/2.

## References

[bb1] Bruker (2001). *SAINT* and *SMART* Bruker AXS Inc., Madison, Wisconsin, USA.

[bb2] Li, J., Meng, X.-G. & Liao, Z.-R. (2005). *Acta Cryst.* E**61**, o3421–o3423.

[bb3] Liu, Y.-C., Ma, J.-F., Hu, N.-H. & Jia, H.-Q. (2003). *Acta Cryst.* E**59**, m361–m363.

[bb4] Santoro, S. W., Joyce, G. F., Sakthivel, K., Gramatikova, S. & Barbas, C. F. (2000). *J. Am. Chem. Soc.* **122**, 2433–2439. 10.1021/ja993688s 11543272

[bb5] Sheldrick, G. M. (1996). *SADABS* University of Göttingen, Germany.

[bb6] Sheldrick, G. M. (2008). *Acta Cryst.* A**64**, 112–122. 10.1107/S0108767307043930 18156677

[bb7] Spek, A. L. (2009). *Acta Cryst.* D**65**, 148–155. 10.1107/S090744490804362X PMC2631630 19171970

[bb8] Sundberg, R. J., Yilmaz, I. & Mente, D. C. (1977). *Inorg. Chem.* **16**, 1470–1476.

[bb9] Xi, Y., Jiang, M., Li, J., Wang, C., Yan, J.-F. & Zhang, F.-X. (2006). *Acta Chim. Sin.* **64**, 1183–1188.

